# Improved assembly procedure of viral RNA genomes amplified with Phi29 polymerase from new generation sequencing data

**DOI:** 10.1186/s40659-016-0099-y

**Published:** 2016-09-07

**Authors:** Nicolas Berthet, Stéphane Descorps-Declère, Andriniaina Andy Nkili-Meyong, Emmanuel Nakouné, Antoine Gessain, Jean-Claude Manuguerra, Mirdad Kazanji

**Affiliations:** 1 Institut Pasteur, Epidemiology and Physiopathology of Oncogenic Viruses, 25 Rue Du Docteur Roux, 75724 Paris, France; 2 Centre National de La Recherche Scientifique, UMR 3569, 25 Rue Du Docteur Roux, 75724 Paris, France; 3 Département Zoonose Et Maladies Emergentes, Syndromes Cliniques Et Virus Associés, Centre International de Recherches Médicales de Franceville (CIRMF), BP769 Franceville, Gabon; 4 Institut Pasteur, Plate-forme de Bioanalyse Génomique, 25 Rue Du Docteur Roux, 75724 Paris, France; 5 Département de Virologie, Institut Pasteur de Bangui, BP 923 Bangui, République Centrafricaine; 6 Unité Environnement Et Risques Infectieux, Institut Pasteur, Cellule D’Intervention Biologique D’Urgence, 25 Rue Du Docteur Roux, 75724 Paris, France

**Keywords:** RNA viral genome, Next generation sequencing, SPAdes, Assembling genome, Amplification with phi29 polymerase

## Abstract

**Background:**

New sequencing technologies have opened the way to the discovery and the characterization of pathogenic viruses in clinical samples. However, the use of these new methods can require an amplification of viral RNA prior to the sequencing. Among all the available methods, the procedure based on the use of Phi29 polymerase produces a huge amount of amplified DNA. However, its major disadvantage is to generate a large number of chimeric sequences which can affect the assembly step. The pre-process method proposed in this study strongly limits the negative impact of chimeric reads in order to obtain the full-length of viral genomes.

**Findings:**

Three different assembly softwares (ABySS, Ray and SPAdes) were tested for their ability to correctly assemble the full-length of viral genomes. Although in all cases, our pre-processed method improved genome assembly, only its combination with the use of SPAdes allowed us to obtain the full-length of the viral genomes tested in one contig.

**Conclusions:**

The proposed pipeline is able to overcome drawbacks due to the generation of chimeric reads during the amplification of viral RNA which considerably improves the assembling of full-length viral genomes.

**Electronic supplementary material:**

The online version of this article (doi:10.1186/s40659-016-0099-y) contains supplementary material, which is available to authorized users.

## Background

Recent improvements in sequencing technologies, referred to as “next-generation” sequencing (NGS), have opened the way in the investigation of infectious etiologies associated to various clinical samples. Indeed, for a decade, this approach has allowed the discovery of unknown and potentially pathogenic viruses in a large set of human samples [1] and it tends to disprove the link between the presence of an unknown infectious agent to some types of cancers [2, 3]. Irrespective of the application, the most crucial and important step regarding our capacity to extract relevant information from NGS sequence data is bioinformatics analysis.

Indeed, after quality control and pre-processing of raw reads, one of the final goals of analysis for some projects concerning pathogen discovery or the detection of specific target organisms, is to generate contigs as large as possible and to assign each sequence present in the sample to a taxon [4]. In several studies, a de novo assembly was directly performed after read trimming followed by an alignment of the contigs using BLAST in order to identify the genotypes present [1–3]. Taxonomic classification based on BLAST, or similar tools such as USEARCH, is common and less time consuming when larger contigs are implied rather than multiple small contigs or singletons. That is why an assembly step is often performed prior to the classification and taxonomic assignation even if this step is not systematically required according to the tool used for viral and bacterial assignation [5].

From a bioinformatics perspective, the step to correctly assemble the reads stemming from the raw data is crucial. In theory, deep sequencing should produce sufficient amounts of data to allow the assembly of large contiguous chunks of genomic DNA, potentially entire viral genomes. However, in practice, it is rarely the case. The assembly of viral data is a difficult task due to several different factors such as a high variability in the coverage or the presence of chimeric reads. Their presence in the raw data could be due to amplification during sample preparation, in particular for RNA viral genomes. Indeed, an amplification of viral RNA using a method based on the Phi29 enzyme produces a huge amount of chimeric reads during the random ligation of all cDNA generated by a random retrotranscription [6]. It is therefore not surprising that these classical tools perform poorly when dealing with RNA viral genomes which were amplified randomly. Here, we present a pre-processing procedure improving the assembly of reads by limiting the impact of chimeric reads data generated from viral RNA amplified with Phi29 polymerase.

## Methods

### Virus isolation, extraction of RNA, random amplification and high-throughput sequencing

A strain of the Middelburg virus (MIDV-ArTB-5290) and of a Mengovirus (AnrB-3741), isolated during arthropod surveillance in the Central African Republic in 1984 from *Amblyomma variegatum* and in 1983 from *Tatera* sp, a species belonging to rodents (Gerbilinae), respectively, were amplified by serial passage in the brain of new-born mice. After several passages, the brains were homogenized and centrifuged before a lyophilisation of each supernatant. RNA extraction was performed using the QIAmp viral RNA minikit according to the manufacturer’s instructions from resuspended lyophilizates in sterile water. Extracted RNAs were treated with Turbo DNAse (Invitrogen Inc., Carlsbad, CA) in order to remove contaminating DNA (i.e. host genome of *Mus musculus)* and then retrotranscribed into cDNA using SuperScript III reverse transcriptase (Invitrogen Inc., Carlsbad, CA) and random hexamer primers. This cDNA was amplified based on a universal and “unbiased” method with a phi29 enzyme as previously described [6]. The generated DNA fragments were used to construct a genomic library with the TruSeq DNA sample prep kit V2 (Illumina) according to the manufacturer’s recommendations. The Illumina Sequencing was conducted using HiSeq 2000.

### Bioinformatic analysis

**Fig. 1:**
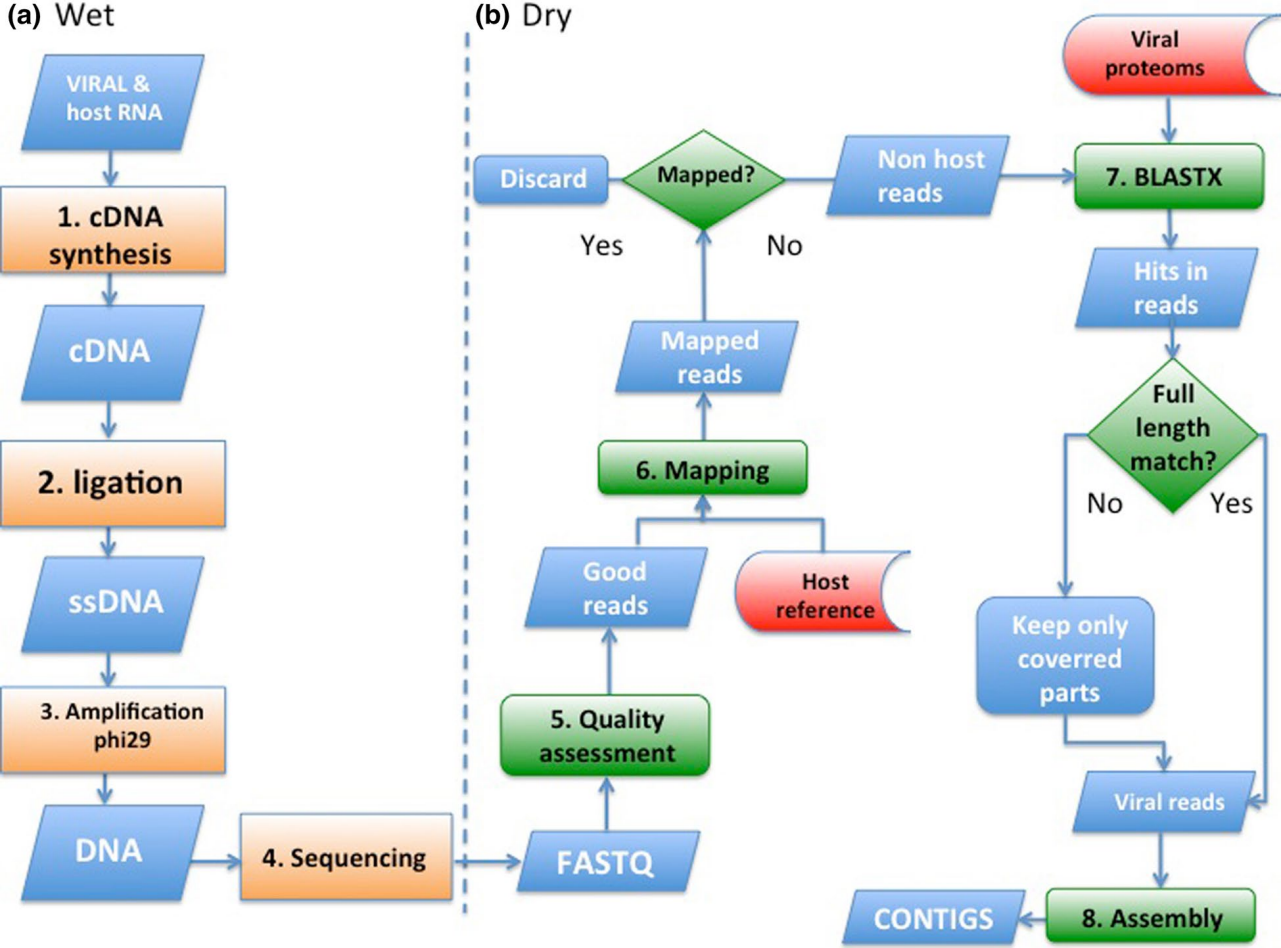
Figure describing the main steps of retrotranscription, amplification of RNA and sequencing (**a**) and the viral reads’ filtering method (**b**). This method is divided in different parts. The first part obtains all reads in Fasta format after different types of filtration steps. The second step aims at selecting only the viral part in each read using a similarity-based approach. Finally, the last step is to perform assembly using different algorithms with targeted sequences. *HTS* high throughput sequencing; *cDNA* complementary DNA; *ssDNA* single strand DNA

The quality of the reads was initially assessed by FastQC. The mouse genome sequence was filtered by mapping the selected reads on the *Mus musculus* Mn10 sequence using Bowtie 2.0 software with the “very sensitive” flag option [7]. All remaining reads corresponding to viral sequences were obtained based on “similarity-based” approach and used BLASTN and BLASTX with a defined number of targeted sequences available in sequence databanks (L22089, DQ294633.1 and KF680222.1). All viral reads were selected according to the percentage of identity (a minimum of 75 %) between the reads and reference sequences and a minimum alignment length of 60 bases including indel. In order to improve the assemblage quality of viral genomes, only the region of each read matching BLAST results was selected and kept (Fig. 1). This way, all non-viral sequences potentially associated with a viral sequence inside the same read generated during the retrotranscription step were removed. The selected reads were assembled with different software, such as ABySS, Ray and SPAdes (version 3.0; 3.5 and 3.6) with different *k* values used to build the Bruijn graph [8, 9]. All genome assemblies were evaluated using the QUAST tool such as the number of obtained contigs, the size of the largest contig, the N50 and L50 and finally, the coverage of the genome obtained [10]. The proportion of reads which unmapped on generated contig(s) for each set of data was determined by mapping, by using Bowtie 2.0 software with the “very sensitive” flag option and “End to End” as the alignment type in the Geneious R9 software. All chimeric reads were identified from a tabular output of a BLAST generated file which contained matching positions from reads against BLAST hits. A read was considered to be chimeric if its entire sequence did not belong to the alignment.

### Assignation of the viral chimeric fragments

The taxonomic assignation of each viral chimeric fragment was identified from the tabulated outputs of BLAST. For each alignment or high-scoring segment pairs (HSP) in the latter, the part of each viral read which did not align with the viral reference, was considered as a chimeric fragment. A python script allowed to retrieve such chimeric fragments and to plot their size distribution. They were then selected with a threshold of 30 bp minimum size and aligned with BLAST against viral references.

### Graph complexity assessment

The complexity of graphs resulting from both targeted and untargeted reads was assessed according to the BEST theorem. The number of operations for counting eulerian paths was based on this following associated formula ec(G) = t_w_(G)∏_v∈V_(deg(v)−1)! [11].

### Phylogenetic analysis

A total of 9 complete genome sequences of Mengoviruses/EMCV that are available in GenBank were aligned using the ClustalW algorithm in Geneious software for Mac (Geneious version R9—http://www.geneious.com). The Bayesian Markov chain method in MrBayes (version 3.2) was used to build the phylogenetic trees using two runs of four chains with 1 million generations, with a burn-in rate of 25 % and the GTR + G + I nucleotide substitution model [12].

### Statistical analysis

Statistical analysis was performed using Excel software from Microsoft Office for Mac version 14.0. The Fisher test was used to compare the percentage of unmapped reads on contig(s) between sets of sequencing data.

### Nucleotide sequence accession numbers

The whole-genome sequences are available in the DDBJ/EMBL/GenBank database under accession number KU955338.

## Results

### Global analysis of sequencing data

A mean depth of 10 × 10^6^ single reads of 100 nucleotides (nt) size was generated for each sample. According to the sample, about 33 to 44 % of all reads were filtered on *Mus musculus* Mm 10 reference genome by mapping with Bowtie 2.0 software. In the end, the reads’ proportion retained after host read removal and quality trimming ranged from 53 to 63 %. The mean trimmed read length ranged from 87.13 to 93.75 and the Phred score was 36 for each sample (Table 1). A total of 136,903; 357,760 and 495,356 reads representing 2.1, 5.46 and 6.1 % of trimmed reads (for ANRB3741, ARTB5290 and ARB19017 respectively) were selected through the use of BLASTN/X tools from a sample group of available sequences in GenBank (table in supplementary data). The quality of the assembly of viral sequences was compared based on the use of different assemblers. The number of contigs ranged from 35 to 20,618 depending on the individually assembler tested (Table 2). The size of the largest contig varied between 331 and 3492 for SPAdes v3.5 (ARTB5290) and SPAdes v3.0 (ARB19017), respectively. The numbers of contigs generated with Ray were less numerous than those obtained with ABySS (12 to 35 versus 5908 to 20,618, respectively). However, the average of the largest contig size and N50 value was higher with Ray than ABySS (559 versus 124, respectively) (Table 2). Except for the ANRB3741 sample, the results for all the parameters of assembly were different according to the version of SPAdes tested (3.0 versus 3.5/3.6). Whatever the tested assembler, no full-length viral genome was directly obtained with untargeted sequences.

**Table 1:** Overview of sequencing data

	Middelburg ArTB 5290	Mengovirus AnrB 3741	Mengovirus ArB 19017
Total number of reads	11,875,121	11,925,315	12,708,896
Mean read length (bp)	101	101	101
*Mus musculus* reads removal by mapping	4,951,166 (41.69 %)	5,207,943 (43.67 %)	4,218,708 (33.35 %)
Trimmed reads (after host removal)	6,549,596 (58.31 %)	6,372,036 (53.43 %)	8,097,092 (63.71 %)
Mean trimmed read length (bp)	93.75	90.49	87.13
Mean Phred score	36	36	36
Total number of viral reads	357,760 (5.46 %)	136,903 (2.1 %)	495,356 (6.1 %)

**Table 2:** Assembly of Mengovirus and MIDV genomes with different assembler software with targeted and untargeted sequences obtained after selection using similarity-based approach

		ArTB 5290		ArB 19017		AnrB 3741	
		Targeted	Untargeted	Targeted	Untargeted	Targeted	Untargeted
ABYSS	Contigs (≥50 bp)	2463	11,822	24	20,618	15	5905
	Contigs (≥1000 bp)	3	0	2	0	1	0
	Largest contig	1807	372	3219	195	7025	266
	Mean length	88	84	576	82	392	82
	N50	1185	124	2574	124	7025	124
	L50	3	1501	1	2218	1	599
Ray	Contigs (≥50 bp)	2	35	3	12	4	28
	Contigs (≥1000 bp)	2	0	2	2	2	0
	Largest contig	6906	837	4088	1873	4360	552
	Mean length	4844	284	2013	210	2571	523
	N50	6906	575	4088	1102	3745	552
	L50	1	2	1	2	1	1
SPAdes v3.0	Contigs (≥50 bp)	2	371	1	16	1	322
	Contigs (≥1000 bp)	1	2	1	2	1	0
	Largest contig	11,468	1065	7548	3492	7562	951
	Mean length	5789	145	7562	127	7548	110
	N50	11,468	892	7548	3492	7562	951
	L50	1	3	1	1	1	1
SPAdes v3.5/SPAdes v3.6	Contigs (≥50 bp)	5	10,435	7	569	2	322
	Contigs (≥1000 bp)	1	0	1	0	1	0
	Largest contig	11,314	331	7548	396	7548	951
	Mean length	2359	100	3896	127	1174	110
	N50	11,314	129	7548	141	7548	951
	L50	1	2492	1	113	1	1

The reads whose regions of the viral sequences were selected within the reads were named «Targeted Sequences» or TS, whereas the untreated sequences were named «Untargeted Sequences» or US

### Assembly after chimeric part removal from reads

For each set of sequencing data, only the portion corresponding to a viral sequence was selected within the reads according to the BLAST result. They were named «targeted sequences» or TS. As before, the numbers of contigs ranged from 1 to 2463 according to the assembler used (Table 2). Moreover, whatever the studied parameters on the size of generated contigs, their values were significantly higher in comparison with untargeted sequences (Table 2). Indeed, the size of the largest contig varied between 1807 and 11,468 respectively for ABySS and SPAdes v3.0 (ARTB5290). However differences were observed between the tested assemblers. Indeed, SPAdes v3.0 was able to obtain one contig that covers the full length of the viral genome and Ray two contigs overlapped by 15 bases. On the other hand, ABySS delivered a higher number of contigs whose sizes as well as the genome’s coverage were lower compared to those obtained with Ray and SPAdes v3.0 (Table 2). Two recent updates of SPAdes (version 3.5 and 3.6) were also tested with the same set of data. Unfortunately, similar results were obtained and these novel versions did not improve the quality of assembling, whatever the set of sequences tested.

### Impact of chimeric reads on the assembly process

The influence of chimeric reads on the assembly process was evaluated through several parameters such as their proportion in each set of data or outputs of graphs provided by the assembler. The proportion of these reads, which contained viral and non-viral portions, represented 35.89, 50.72 and 64.13 % of the reads for the three samples tested (ArNB-3741, ARB5290 and ARB19017, respectively). Analysis of the chimeric part of each read showed that fragment size ranged from 1 to 73 bases with an average size from 16 to 32.7 bases (Additional file 1: Fig. S1). The proportion of chimeric fragments whose size was superior to 30 bases, ranged from 4.43 to 72 % according to the considered set of data (Additional file 2: Table S1). After BLAST analysis of these fragments against viral genomes, only 28.8, 80.3 and 97.7 % were correctly aligned on reference sequences for AnrB-3741, ArB19017 and ArB5290, respectively (Additional file 2: Table S1). After the assembly process, the proportion of unmapped reads on different contigs previously generated was significantly different between targeted and untargeted sequences whatever the assembler used (p value 5.10e−6). Indeed, values ranged from 0.5 to 6.99 % and from 34.1 to 71.4 % for targeted and untargeted sequences, respectively. This proportion of difference of unmapped reads was also significant for each assembler individually tested (ABySS and SPAdes 3.0 and 3.5/3.6) except for Ray (p value 0.14) (Table 3). Based on a second approach with graph generation, only Ray provided this output with several parameters such as the number of vertices and edges for each set of data. These numbers for untargeted reads assembly were two to four times higher than those for targeted sequences or with assemblies whose chimeric parts were removed (Table 4). Moreover, according to the formula based on the BEST theorem used for counting eulerian paths, all graphs with untargeted sequences showed a greater complexity in comparison with graphs from reads with removed chimeric parts. Indeed, the first and second factors of the product from BEST theorem’s formula depend on the number of edges and of vertices, respectively. Therefore, the increase of these values implied a rise in the complexity for untargeted reads graphs. Finally, all the set of data without chimeric reads (not only the chimeric parts but the whole reads) were mapped again on all the contigs obtained previously with targeted and untargeted sequences. The percentage of unmapped reads was significantly higher (1.8 *versus* 32.53 %, p value 1.8.10e−4) for sets of reads against contigs from targeted and untargeted sequences, respectively (data not shown). The initial presence of chimeric parts inside reads induced a huge number of small-sized contigs as well as the generation of chimeric contigs whatever the assembler used.

**Table 3:** Percentage of reads which unmapped on contigs generated with different assemblers

		Middelburg ArTB 5290	Mengovirus AnrB 3741	Mengovirus ArB 19017	p value^a^
Abyss	Targeted sequences	6.99 %	2.64 %	2.25 %	0.04
	Untargeted sequences	40.59 %	36.93 %	69.78 %	
Ray	Targeted sequences	6.76 %	0.92 %	2.26 %	0.14
	Untargeted sequences	53.84 %	51.5 %	71.41 %	
SPAdes 3.0	Targeted sequences	0.57 %	0.82 %	0.65 %	0.001
	Untargeted sequences	47.39 %	55.87 %	60.02 %	
SPAdes 3.5/3.6	Targeted sequences	3.05 %	0.6 %	0.48 %	0.02
	Untargeted sequences	34.12 %	55.54 %	60.02 %	
p value^a^		0.13	0.003	0.01	5.10e−6

^a^ Determined according to the Fisher Test

**Table 4:** Graph features from targeted, untargeted and chimeric-part-removed reads

	Parameters	
	Number of vertices	Number of edges
MIDV ARB5290		
Targeted sequences	220,838	438,926
Untargeted sequences	719,848	1,442,274
Chimeric reads removal	N.A	N.A
Mengo 19017		
Targeted sequences	790,086	1,572,606
Untargeted sequences	1,411,912	2,803,374
Chimeric reads removal	573,848	1,143,146
Mengo 3741		
Targeted sequences	135,440	264,970
Untargeted sequences	534,694	1,052,622
Chimeric reads removal	58,452	115,682

### Phylogenetic analysis of a novel variant of Mengovirus isolated in the Central African Republic

**Fig. 2:**
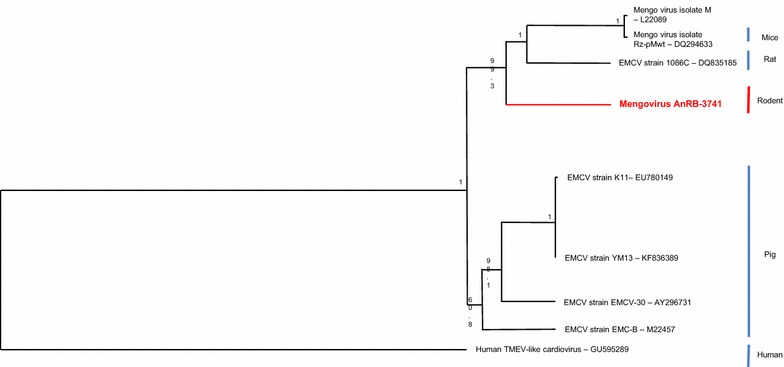
Phylogenetic tree of mengo and encephalomyocarditis viruses. Phylogenetic analysis was based on nucleic acid sequences of the whole genomes of mengo and encephalomyocarditis viruses from the NCBI database

The Mengovirus belongs to the genus *Cardiovirus* and the Picornaviridae family. It was isolated for the first time in 1948 in Uganda from a rhesus monkey which had developed hind limb paralysis. Genomic analysis of 7717 nucleotides for the strain ArNB-3741 showed the typical organization of the genome of the Mengovirus with one ORF encoding a polyprotein of 2293 amino acid flancked by two UTR in 5′ and 3′. Our genome of the Mengovirus shares 79.7 and 94.5 % at nucleic and amino acids levels with the sequence of the EMC virus (1086C), the closest strain. The phylogenetic tree based on the polyprotein sequence showed that our strain of Mengovirus isolated in the CAR belongs to the group of mengo/EMC viruses isolated from different species of rodents (mice and rats) (Fig. 2).

## Discussion and conclusions

In this study, we describe an efficient procedure to improve the assembly of amplified RNA viral genomes based on the use of Phi29 polymerase. This method of amplification generated a large amount of amplified DNA. However, the major disadvantages are a stochastic amplification bias and/or the generation of chimeric fragments. Indeed, detailed analysis showed heterogeneity either in the size of the chimeric fragments or in the nature of these sequences even if only fragments superior to 30 bp were analysed. These fragments have proven to be troublesome because they can disturb the assembly process. Indeed, these chimeric reads, whatever heterogeneity observed in their numbers, their sizes or their nature of sequences, will affect the De Bruijn graph construction by putting “false” k-mers in it. This can lead to count wrong paths in the graph, resulting in either finding false Eulerian paths if the “false” k-mers are shared among other reads, or yielding small contigs. Although these assembly tools based on De Bruijn Graphs are widely used, the issue with the OLC method is pretty much the same when it comes to finding the overlap. Indeed, reads displaying chimeric parts won’t extend the assembly because the “false” overlap may not be present in other reads. The result will also be a poor quality assembly with multiple small contigs that failed to be extended. Basically, every assembler is affected the same way by chimeric reads because they rely on overlapping information. Some recent assemblers such as MetaVelvet or SPAdes contain a chimeric read removal module which is built on heuristics based on coverage difference [8, 13]. Unfortunately, our results tend to confirm that these chimeric junctions generated during the amplification step are not detected by these heuristics. However, current chimeric issues can be dealt with specific tools, which could detect said flawed sequences such as CHIMERA_CHECK, Pintail and Mallard [14–16]. But these tools are limited by the use of trusted chimera-free reference sequences. On the other hand, other tools such as Perseus or ChimeraSlayer are based on alignments [17, 18]. According to the kind of samples tested, the chimeric reads removal approach is probably not the best answer because it often results in a low amount of employable unique read pairs for assembly [19].

In the field of metagenomics, one of the first priorities in the analyses of the generated data is being able to assign contigs or singletons to a taxon, either by a sequence homology by searching for the signature an infectious agent (GOTTCHA), or by the use of another algorithm such as Kraken [4, 20]. Even if the size of the sequence which we aim to assign is not a limiting criteria, the bigger its size, the easier the assignation will be, especially if the divergence with a reference sequence is great. A de novo preliminary assembly step is usually achieved regardless of the method of preparation of nucleic acids and their prior amplification or non-amplification, in order to, on one hand, increase the size of the sequences to assign, and on the other hand, diminish their number. In this situation in metagenomics, when obtaining the entire genome of a viral agent is less sought and very unlikely, obtaining very small-sized contigs after an assembly is sufficient to allow their assignation and to gather them in viral families [21]. However, in some cases, after identification by a metagenomic approach, obtaining a viral pathogen’s entire genome is important for a better molecular characterization of the identified viral strain. When the divergence with a reference genome is low, the sequence can be easily achieved by mapping, for example in the case of the Middelburg virus in this study [22]. However, in the case of a virus whose sequence is strongly divergent compared to the closest reference sequence (for instance in this study with our Mengovirus AnRB which presents about 20 % of divergence on the nucleic level), the approach by mapping is not adapted except at the level of the slightly divergent genomic region. In this case, the finalization of the genome can only be obtained either with the combination of the Sanger sequencing to complete the missing regions of the genome initially obtained through several contigs, or by improving the de novo assembly in order to obtain longer contigs covering the whole genome.

In conclusion, the elimination of the reads or of the chimeric portions in the reads generated during the RNA amplification steps are the key to an improvement in the assembling of the sequences in order to obtain the whole genome of the considered RNA virus. This procedure using a pipeline of tools is able to overcome potential drawbacks due to the amplification methods and yields a much better assemblage of viral genomes even when no close reference sequence is available.

## Additional files

10.1186/s40659-016-0099-ySize distribution of chimeric portions.
10.1186/s40659-016-0099-yFeatures of chimeric fragments. The x-axis corresponds to the size of chimeric sequences whereas the Y-axis to the number of chimeric sequences.

## Abbreviations

NGS: new generation sequencing
cDNA: complementary DNA
BLAST: basic local alignment search tools
TS: targeted sequence
US: untargeted sequence
BEST: Bruijn, van Aardenne-Ehrenfest, Smith and Tutte
EMCV: encephalomyocarditis virus
OLC: overlap layout consensus
