# Supplementary figures and images for: Improved assembly procedure of viral RNA genomes amplified with Phi29 polymerase from new generation sequencing data
Source: Biol Res. 2016 Sep 7;49(1):39. doi: 10.1186/s40659-016-0099-y (PMC5015205; doi:10.1186/s40659-016-0099-y)

## Slide 1
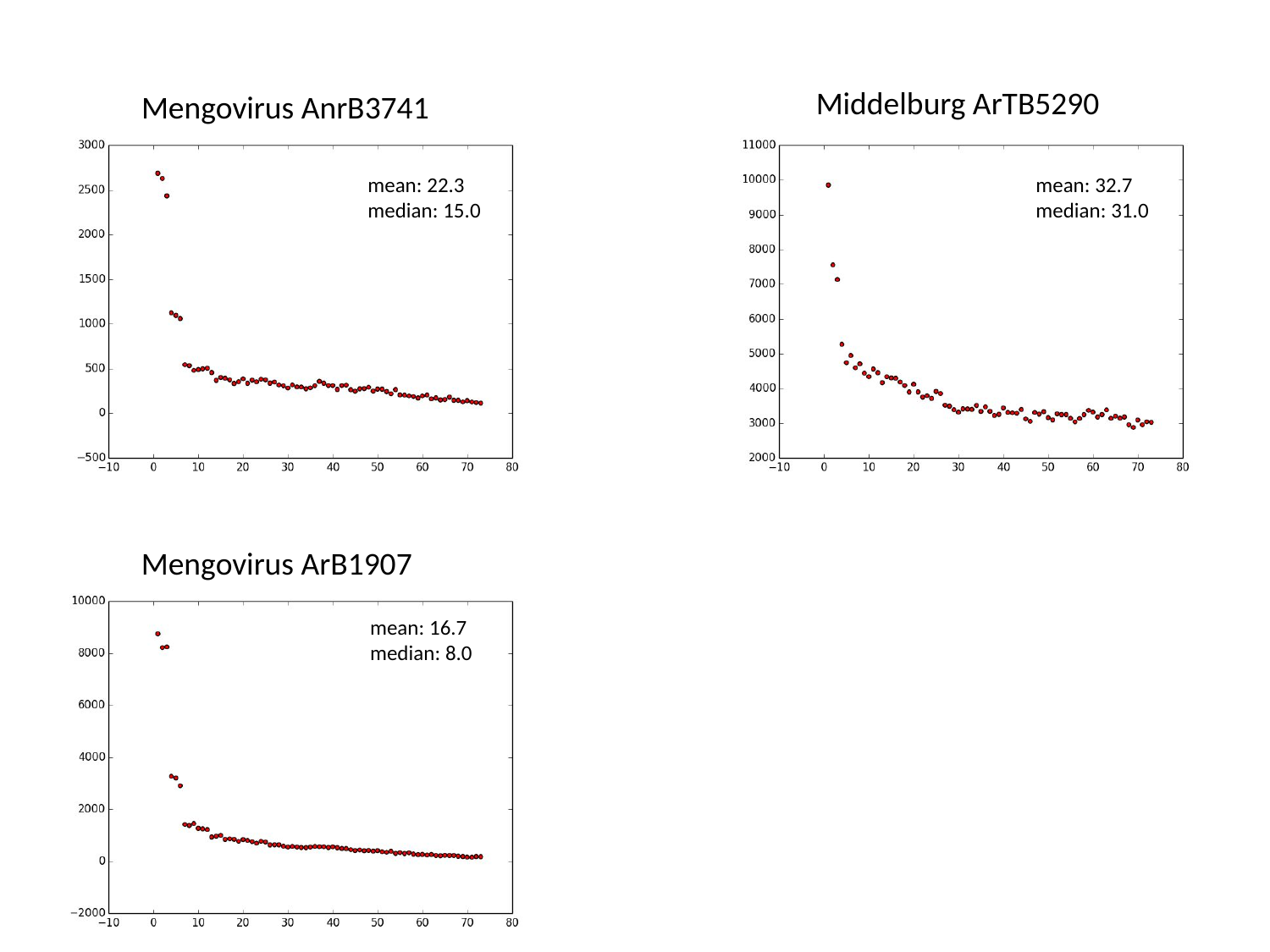

Middelburg ArTB5290
Mengovirus AnrB3741
mean: 22.3
median: 15.0
mean: 32.7
median: 31.0
Mengovirus ArB1907
mean: 16.7
median: 8.0

Supplement: Supplementary file 1 — 10.1186/s40659-016-0099-ySize distribution of chimeric portions. [file 40659_2016_99_MOESM1_ESM.pptx]
