# Supplementary material for: Improved assembly procedure of viral RNA genomes amplified with Phi29 polymerase from new generation sequencing data
Source: Biol Res. 2016 Sep 7;49(1):39. doi: 10.1186/s40659-016-0099-y (PMC5015205; doi:10.1186/s40659-016-0099-y)
Supplement: Supplementary file 2 — 10.1186/s40659-016-0099-yFeatures of chimeric fragments. The x-axis corresponds to the size of chimeric sequences whereas the Y-axis to the number of chimeric sequences. [file 40659_2016_99_MOESM2_ESM.docx]

**Table S1. Features of chimeric fragments**

|  | **Middelburg**  **ArTB 5290** | **Mengovirus**  **AnrB 3741** | **Mengovirus**  **ArB 19017** |
| --- | --- | --- | --- |
| Proportion of chimeric reads / total number of reads | 50.7% | 35.8% | 64.1% |
| Proportion of chimeric part >30 bp / total number of chimeric reads | 72.0% | 19.9% | 4.43% |
| Proportion of viral chimeric part / total number of chimeric reads (>30bp) | 97.7% | 28.8% | 80.3% |
